# Bicomponent Cutaneous Cell Therapy for Early Burn Care: Manufacturing Homogeneity and Epidermis-Structuring Functions of Clinical Grade FE002-SK2 Allogeneic Dermal Progenitor Fibroblasts

**DOI:** 10.3390/pharmaceutics17060692

**Published:** 2025-05-24

**Authors:** Xi Chen, Nathalie Hirt-Burri, Corinne Scaletta, Alexis E. Laurent, Lee Ann Applegate

**Affiliations:** 1 Regenerative Therapy Unit, Lausanne University Hospital, University of Lausanne, CH-1066 Epalinges, Switzerland; xi.chen.1@unil.ch (X.C.); nathalie.burri@chuv.ch (N.H.-B.); corinne.scaletta@chuv.ch (C.S.); 2 Manufacturing Department, TEC-PHARMA SA, CH-1038 Bercher, Switzerland; 3 Center for Applied Biotechnology and Molecular Medicine, University of Zurich, CH-8057 Zurich, Switzerland; 4 Oxford OSCAR Suzhou Center, Oxford University, Suzhou 215123, China

**Keywords:** burns, cell banking, cell therapy, chronic wounds, extracellular matrix, fetal skin, fibroblasts, mesenchymal stem cells, progenitor cells, wound healing

## Abstract

**Background:** The extracellular matrix (ECM), primarily composed of collagen and elastin synthesized by dermal fibroblasts, is critical for mesenchymal tissue integrity. Fibroblast phenotypes vary significantly with the anatomical location and developmental stage. Fetal skin, particularly prior to 14 weeks of gestation, exhibits a simplified structure compared to adult skin, characterized by a thin, loose dermal matrix and a single-layered epithelium. **Objectives:** This study aimed to characterize and functionally compare homogenous progenitor fetal fibroblast (PFF) populations derived from 14-week-old fetal skin with fibroblasts isolated from adult burn patients. **Methods:** We evaluated the proliferative capacity, collagen synthesis, and differentiation potential (adipogenesis and osteogenesis) of PFF and adult burn patient fibroblasts. Furthermore, we assessed their ability to support skin regeneration using a de-epidermized dermis (DED) model seeded with both PFF and patient-derived keratinocytes. The stability of PFF characteristics was monitored across multiple passages (P5–P12). **Results:** PFF demonstrated a 2–4-fold increase in proliferation rate and a 30–50% enhancement in collagen production in vitro compared to adult fibroblasts. Notably, PFF exhibited a consistent lack of adipogenic and osteogenic differentiation, an attribute distinct from adult fibroblasts. In the DED model, PFF, even at a low fibroblast-to-keratinocyte ratio (1:5), effectively facilitated the formation of well-organized skin structures, including rete ridges, surpassing the performance of adult fibroblasts and adipose-derived cells. These properties remained stable over multiple passages. **Conclusions:** The unique attributes of PFF, likely attributable to the simplified microenvironment (i.e., collagen organization) of developing fetal tissue, positions them as a promising source for cell-based therapies. Their inherent high collagen synthesis capacity is particularly advantageous for wound healing applications. Consequently, PFF represent a consistent and readily available resource for developing “off-the-freezer” cutaneous cell therapies, potentially enabling accelerated and improved treatment of severe burn injuries.

## 1. Introduction

Cell therapies for wound healing have undergone substantial advancements, encompassing a diverse array of cell types, origins, and formulations. Early therapies, pioneered for burn patients, relied heavily on autologous skin biopsies [1,2,3]. These protocols typically involved the ex vivo expansion of both autologous keratinocytes and fibroblasts, subsequently utilized in cell therapies or tissue-engineered constructs, with or without further in vitro manipulation [2]. Notably, the treatment of extensive wounds necessitates significant cell expansion to ensure adequate therapeutic material availability. Consequently, preparations such as cultured epithelial autografts (CEA) and cultured dermal–epidermal autografts (CDEA) have been clinically employed for decades, demonstrating efficacy in providing life-saving skin coverage [1,2,3,4,5,6,7,8,9].

While CEAs effectively cover large burn wounds, offering crucial epidermal reconstitution, they often yield suboptimal aesthetic outcomes due to the absence of concurrent dermal regeneration. However, when integrated with mesh grafts (where available), CEAs can ameliorate scar appearance by minimizing mesh patterns and accelerating epithelialization [2,3,4,5,6,7,8,9]. The success of these therapies in improving survival rates for burn patients during the 1980s catalyzed the application of tissue engineering principles to a broader spectrum of skin pathologies, including vitiligo, blistering diseases (e.g., epidermolysis bullosa), and chronic ulcers. This expansion has prompted the exploration of diverse cell sources for skin reconstruction [3].

Currently, a wide range of cell sources are under investigation in clinical trials globally, aiming to identify optimal candidates for wound and burn management. These studies prioritize the development of therapies capable of rapid cell expansion for efficient treatment delivery. Of note, these investigations encompass stem cells derived from bone marrow (BM-MSCs) and adipose tissue (AD-MSCs or ASCs), as well as stem cells from umbilical cord, placenta, peripheral blood, hair follicles, and even patient-derived sources such as keratinocyte progenitors and excised burn skin stem cells [6,10,11,12,13,14,15,16,17,18,19].

A meta-analysis of preclinical animal studies suggested that hair follicle stem cells exhibit promising potential for burn wound healing. However, the attainment of sufficient cell quantities for large-surface-area burns remains a significant hurdle [10]. Other meta-analyses have documented the historical utilization of BM-MSCs, supported by studies highlighting their capacity for cell proliferation, collagen synthesis, and angiogenesis [11]. Nevertheless, challenges associated with BM-MSC extraction quality and yield have fostered interest in alternative sources like ASCs, which offer more accessible and abundant cell populations [11].

In general, stem cell therapies for burn management have demonstrated promising outcomes, with diverse mechanisms of action elucidated. These cell sources (autologous, allogeneic, or xenogeneic) can be harvested at various developmental stages [19,20,21,22,23,24,25,26,27,28,29]. Consequently, numerous advancements in cell therapies for burn and wound management have been reported. Key observed benefits include accelerated wound closure, enhanced skin regeneration with increased soft tissue formation and collagen deposition, reduced scar tissue formation, improved pain management, attenuated inflammatory responses, and even systemic effects on metabolism, inflammation, and immunomodulation [13,15].

However, the widespread clinical adoption of cell therapies has been impeded by several factors related to cell sources, including their differentiation potential towards alternative lineages, the risk of cell fusion, and the optimization of interactions between growth factors, biomaterials, and delivery systems post-transplantation. Recently, cell-free therapies (e.g., exosomes, secretome, or cell-conditioned media) have emerged as a promising alternative, leveraging paracrine signaling for wound healing [15,17]. Nevertheless, from a manufacturing standpoint, these therapies present increased complexity due to intricate processing and purification requirements [15,17]. Thus, while cell-free therapies offer distinct advantages, classical cell therapies provide sustained and comprehensive delivery of essential growth factors, cytokines, and proteins, more closely mimicking the dynamic needs of the local wound microenvironment. Given that intact cells naturally contain specific cellular byproducts, further preclinical and clinical research is warranted to assess the efficacy of processed sub-fractions compared to the utilization of whole cells [29].

Clinical consensus generally supports the notion that skin repair is optimized when fibroblasts and keratinocytes are co-delivered [30,31,32,33,34]. This was experimentally validated in a porcine wound model, where compartmentalized chambers were used to evaluate the effects of different cell combinations [32]. The addition of fibroblasts to keratinocyte suspensions was observed to enhance epithelialization within the granulation tissue, facilitating cell adhesion and migration [32]. While the precise mechanisms underlying the synergistic interactions between fibroblasts and keratinocytes remain incompletely understood, they are believed to involve fibroblast-mediated growth stimulation and improved keratinocyte adhesion to the prepared wound bed, facilitated by integrin receptor interactions with the basal membrane [32]. Other autologous cell sources, such as out-root sheath keratinocytes, were explored in early technologies (e.g., Modex) and clinical trials for chronic wounds [35,36]. However, the commercialization of these treatments encountered substantial challenges due to high production costs, stringent quality assurance demands, and logistical complexities associated with patient-specific therapies. Consequently, many companies transitioned towards the development of allogeneic cell therapy products [35,36].

Our translational research group has successfully integrated allogeneic PFF into clinical care for well over two decades [37,38,39,40,41,42]. We have demonstrated the utility of these cells for early burn treatment, enabling initial wound coverage within 4–5 days post-burn, analogous to temporary dressings (e.g., cadaver skin, porcine skin) [43,44,45]. Furthermore, PFF can serve as a dermal substrate for subsequent delivery of patient-derived keratinocytes, effectively generating a CDEA with two cell types. Given the ready preparation and storage capabilities of PFF, they can be combined with patient keratinocytes for early initiation of bicomponent cell therapies [45]. This approach facilitates the extemporaneous utilization of available frozen cell stocks, particularly critical for severe burn patients (50–90% TBSA), bypassing the 40–60 day waiting period associated with conventional CDEA manufacturing. Given the long-standing safe clinical application of PFFs, various strategies can be explored to incorporate these cells with autologous keratinocytes, enabling earlier initiation of patient-specific cell therapies [37,44]. This approach has the potential to significantly reduce the time required for complete wound coverage.

This study aimed to investigate the properties of homogenous PFF sources derived from 14-week-old fetal skin. Specifically, we sought to compare the characteristics of PFF with those of primary fibroblasts obtained from adult burn patients. Furthermore, we aimed to assess the potential of PFF to support skin regeneration. Based on the premise that the less complex fetal environment influences cellular development, we hypothesized that PFF would exhibit distinct behavioral and functional characteristics compared to adult fibroblasts. These hypothesized distinctions included higher proliferation rates, increased collagen production, and limited differentiation potential for PFF. Additionally, we hypothesized that PFF would demonstrate superior support for skin regeneration compared to adult fibroblasts within a suitable in vitro model system comprising a dermal template (DED) and primary keratinocytes.

## 2. Materials and Methods

### 2.1. Ethical Compliance for Skin Tissue Procurement

All skin samples utilized in this study were anonymized, meticulously stored, and rigorously logged within the Departmental Biobank (DAL Biobank, Musculoskeletal Medicine Department, Lausanne University Hospital, CHUV), adhering to internal institutional regulations and an approved protocol [BB029DAL]. This biobank operates under strict guidelines to ensure ethical handling and traceability of biological materials.

The study employed human skin biopsies derived from distinct sources. Fetal skin samples were obtained from organ donations between 13 and 18 weeks of gestation, procured under a validated protocol approved by the local State Ethics Committee (University Hospital of Lausanne–CHUV, Ethics Committee Protocol F-34/99 and #62/07). This protocol ensured compliance with Swiss regulations governing progenitor cell transplantation and the associated progenitor cell biobank. These organ donations were appropriately registered within both programs, guaranteeing adherence to legal and ethical standards.

Progenitor cells used come from four fetal organ donation at 14 weeks of gestational age (F-34/99) and the clinical grade FE002- SK2 primary progenitor fibroblast cell bank.

Adult skin fibroblast cells come from biopsies that were collected from non-injured regions of the thigh or buttocks of eight patients presenting with severe burn injuries and one abdominoplasty from an 80-year-old patient. Additionally, ASCs, serving as control cells in differentiation assays, were obtained from a 16-year-old female with a lipoma.

### 2.2. Histology of Human Skin and Immunohistochemistry Processing

For histological processing, human skin samples, representing various developmental stages, eight fetal skin donations (one at 13 weeks, four at 14 weeks, one at 16 weeks and one at 18 weeks of gestation) and one adult abdominal skin were fixed in formalin (Sigma Aldrich, Buchs, Switzerland) and embedded in paraffin (Sigma Aldrich, Buchs, Switzerland) to preserve tissue morphology. Serial sections of 5 µm thickness were then prepared for histological staining with hematoxylin solution (Hematoxylin HTX, Biosystems, Muttenz, Switzerland) and erythrosine (H&E) (Merck, Darmstadt, Germany), enabling visualization of cellular and ECM components.

For nuclear staining, the slides were immersed in a hematoxylin solution for 10 min, facilitating the binding of hematoxylin to nuclear DNA. Subsequently, the slides were subjected to a brief acid alcohol treatment (1% acid alcohol: 99.9% ethanol (Chemie Brunschwig, Basel, Switzerland), 37% hydrochloric acid (Applichem, Darmstadt, Germany), and ultrapure water in a 700:10:290 volumetric ratio) for 5 s to differentiate the staining. Following a 10 min rinse in running water, ensuring a clear blue background, the slides were counterstained with an erythrosine solution (0.25% Erythrosine B; ultrapure water; 37% formaldehyde, Sigma Aldrich, Buchs, Switzerland, in a 2.5 g: 998 mL: 2 mL proportion) to visualize cytoplasmic components. Slides were then rinsed, dehydrated through a graded ethanol series, and mounted with EuKitt mounting medium (Biosystems, Bobingen, Germany) for long-term preservation and microscopic analysis.

For immunohistochemical staining for p63 (p63 [a p53 homolog at 3q27-29] Ab-1, Clone 4A4, Neomarkers, Fremont, CA, USA), fixed tissue sections of 5 µm thickness were utilized for analysis of p63, a key marker for basal keratinocytes, to delineate the epidermis and dermis in fetal skin across various gestational stages. All incubations were performed in a humidified chamber in the dark, unless otherwise specified, to minimize evaporation and light-induced degradation of reagents.

To mitigate non-specific antibody binding, tissue sections were pre-incubated with a blocking solution for 2 h at 25 °C. The blocking solution consisted of phosphate-buffered saline (PBS, Bichsel, Unterseen, Switzerland) supplemented with 5% fetal bovine serum (FBS) (Merck, Darmstadt, Germany), 7% normal goat serum (NGS, Vector Labs, Newark, CA, USA), and 0.1% Triton X-100 (Sigma Aldrich, Buchs, Switzerland), which served to saturate non-specific binding sites. Subsequently, tissue sections were incubated with a primary antibody specific to p63 at a 1:2000 dilution in a staining solution (PBS containing 5% FBS, 5% NGS, and 0.1% Triton X-100) for 30 min.

Following primary antibody incubation, tissue sections were washed three times for 10 min each in PBS to remove unbound antibody. Sections were then incubated with a biotinylated goat anti-rabbit secondary antibody (Vector Laboratories, Burlingame, CA, USA) at a 1:200 dilution in a solution of PBS containing 5% FBS, 1% NGS, and 0.1% Triton X-100 for 3 h at 25 °C, facilitating signal amplification. After four 5 min washes in PBS, sections were treated with Vectastain ABC reagent (Vector, Burlingame, CA, USA) for 3 h at 25 °C, enabling the formation of a biotin-avidin-enzyme complex at the site of antibody binding.

Following Vectastain ABC incubation, sections were washed three times for 10 min each in PBS and then treated with a 3,3′-diaminobenzidine (DAB, Vector Labs, Burlingame, CA, USA) solution (0.5 mg/mL DAB with 0.32 µL 30% H_2_O_2_ added immediately before incubation) for 1–2 min. DAB, in the presence of peroxidase, forms a brown precipitate at the site of antibody binding, indicating p63 expression. The p63 antibody staining was thus visualized as a brown nuclear coloration, allowing for the precise delineation of epidermal and dermal layers.

### 2.3. Primary Cell Culture

Cell sources were anonymized, meticulously stored, and rigorously logged within the DAL Biobank. For this study, cryopreserved cellular materials were procured under the Swiss progenitor cell transplantation program for four fetal organ donations (PFF 1–PFF 4 cell types at 14 weeks gestation) and the clinical grade FE002-SK2 primary progenitor fibroblast cell source. Adult primary fibroblasts from eight severely burned male patients (19, 32, 33, 35, 43, 55 and 85 years) and HaCaT cells were procured from the DAL biobank. Frozen vials were used as cellular starting materials and were serially expanded in vitro to be used. The fibroblast proliferation medium (i.e., for adult and progenitor cells) was composed of high-glucose Dulbecco’s modified Eagle medium (DMEM, Thermo Fisher Scientific, Waltham, MA, USA) supplemented with 2 mM L-glutamine (Thermo Fisher Scientific, Waltham, MA, USA) and 10% FBS. The cultures were maintained in humidified incubators at 37 °C with 5% CO_2_ and the cell proliferation medium was exchanged twice per week. Adult fibroblasts were used at passage 7. Primary progenitor fibroblasts were serially expanded in vitro and were used for experiments at passage levels ranging from 5 to 12. It is to notice that progenitor cells were primary isolated with an outgrowth method instead of enzymatic digestion. Furthermore, papillary and reticular fibroblast subpopulations were isolated from an adult skin sample obtained from an abdominoplasty procedure, stored in the DAL Biobank. For all adult skin biopsies, initial dissection yielded tissue squares of approximately 4 cm^2^. Full-thickness punch biopsies were then performed to separate the upper papillary dermis from the lower reticular dermis, which included a minor portion of the hypodermis. These tissues were processed for cell culture using the same non-enzymatic outgrowth method described above and cultured in the same growth medium (high-glucose DMEM supplemented with 2 mM L-glutamine and 10% FBS). Cell outgrowth of adult skin fibroblasts was observed between 4 and 7 days post-seeding.

ASCs, serving as control cells in differentiation assays, were obtained from a 16-year-old female donor. These tissues were also processed for cell culture using the non-enzymatic outgrowth method. However, the cell proliferation medium contained 5% human platelet lysate instead of FBS, providing a more clinically relevant and potentially less immunogenic growth supplement (hPL; Sexton Biotechnologies, Indianapolis, IN, USA).

### 2.4. Comparative Cell Growth Curves Using the MTT Assay

To assess the proliferative capacity of the isolated cell populations, comparative growth curves were generated using the MTT assay (Thermo Fisher Scientific, Waltham, MA, USA). Cells were maintained in culture by weekly passaging and incubated at 37 °C in a humidified 5% CO_2_ atmosphere within DMEM supplemented with 10% FBS and 4 mM L-glutamine. The culture medium was replenished twice weekly to ensure optimal nutrient availability and pH maintenance.

Upon reaching near-confluency, cells were detached using TrypLE (Life Technologies, Carlsbad, CA, USA), a gentle enzyme-based dissociation reagent, and enumerated using C-Chip Neubauer hemocytometers (NanoEnTek, Seoul, Republic of Korea). For routine culture maintenance, cells were seeded at a standardized viable density of 1.5 × 10^3^ cells/cm^2^ in T75 flasks, ensuring consistent cell population dynamics.

For growth curve determination, cells were seeded in triplicate at a relative viable density of 1.5 × 10^3^ cells/cm^2^ in 96-well cell culture microplates. This seeding density was chosen to ensure exponential growth during the experimental timeframe.

The MTT assay, based on the reduction of 3-(4,5-dimethylthiazol-2-yl)-2,5-diphenyltetrazolium bromide (MTT) into formazan crystals by mitochondrial dehydrogenases, was employed to quantify viable cell numbers. The intensity of the resulting violet formazan precipitate is directly proportional to the number of metabolically active cells. An MTT solution was prepared extemporaneously in PBS at a concentration of 5 mg/mL. For each 96-well plate, 100 µL of MTT solution was carefully added to each well, and the plates were incubated for 3 h at 37 °C under 5% CO_2_. This incubation period allowed for sufficient formazan formation within viable cells.

Following incubation, the MTT solution was aspirated to prevent interference with subsequent steps. Then, 100 µL of MTT solvent (a 1:24 volumetric ratio of 1 N hydrochloric acid (HCl, Chemie Brunschwig, Basel, Switzerland) to isopropanol (Merck, Darmstadt, Germany) was added to each well to dissolve the formazan crystals. The absorbance of the resulting solution, directly correlating with the amount of formazan formed, was measured colorimetrically at a wavelength of 595 nm using a Varioskan LUX spectrophotometer (Thermo Fisher Scientific, Waltham, MA, USA). Cell proliferation curves were then constructed based on the obtained absorbance values, reflecting the relative number of viable cells at each time point.

### 2.5. Adipogenic Differentiation (Oil Red O) Assessment Methodology

To evaluate the adipogenic differentiation potential of the fibroblast sources, cells were seeded in 12-well culture plates at a density of 10^3^ cells/cm^2^ in their respective complete growth media (DMEM supplemented with 5% hPL for ASC controls and DMEM supplemented with 10% FBS for fibroblasts). The plated cells were incubated at 37 °C in a humidified 5% CO_2_ atmosphere for one week, allowing for cell attachment and initial proliferation. ASCs, serving as control cells in differentiation assays, were obtained from a 16-year-old female donor.

Following the initial week of culture, adipogenic differentiation was induced by replacing the complete growth medium with 1 mL of adipogenic induction medium. Simultaneously, control wells received 1 mL of complete medium supplemented with FBS. The adipogenic induction medium was formulated to promote adipocyte differentiation and consisted of the following: basal medium (DMEM with 10% FBS, 8.70 mL), 100 µL insulin-transferrin-selenium (ITS, Sigma Aldrich, Buchs, Switzerland), 10 µL dexamethasone (1 mM stock, Sigma Aldrich, Buchs, Switzerland), 10 µL indomethacin (100 mM, Sigma Aldrich, Buchs, Switzerland), and 10 µL 3-isobutyl-1-methylxanthine (IBMX) (100 mM, Sigma Aldrich, Buchs, Switzerland). These components collectively stimulate adipogenesis by activating key transcription factors and signaling pathways involved in lipid accumulation. The media in both induced and control wells were exchanged twice weekly to maintain consistent induction conditions.

After 7 days of incubation, preliminary morphological changes were documented using an inverted microscope (Olympus IX81, Olympus, Tokyo, Japan). Oil Red O staining, a lipophilic dye that stains neutral triglycerides and lipids, was performed at both 7 and 14 days for control wells and at 14 days for induced wells to assess adipocyte differentiation.

For staining, wells were initially washed with deionized water to remove residual media. Subsequently, 1 mL of a working solution of Oil Red O (0.18% Oil Red O in 99% isopropanol, Sigma Aldrich, Buchs, Switzerland) was added to each well. The plates were incubated with gentle agitation for 15 min, facilitating the uptake of Oil Red O by intracellular lipid droplets. Following incubation, wells were washed twice with deionized water to remove unbound dye. Finally, 1 mL of deionized water was added to each well, and the plates were stored at 4 °C until imaging.

Imaging was performed using an Olympus IX81 microscope with a 10× objective lens. Images were captured in triplicate from different areas within each well to ensure representative sampling and minimize variability. PFF cells were assessed across at least four passages (P4–P12), while adult fibroblasts were analyzed over 3–4 consecutive passages, each performed in triplicate, to evaluate the consistency of adipogenic differentiation potential across different cell populations and passages.

### 2.6. Osteogenesis Differentiation (Alizarin Red and Von Kossa) Methodology

To evaluate the osteogenic differentiation potential of the cell populations, cells were seeded at a density of 10^4^ cells/cm^2^ in 12-well plates and incubated for 24 h at 37 °C in a humidified 5% CO_2_ atmosphere within a standard cell culture medium (DMEM supplemented with 10% FBS and 1% L-glutamine). This initial incubation period allowed for cell attachment and even distribution across the well surface. ASCs, serving as control cells in differentiation assays, were obtained from a 16-year-old female donor.

Upon reaching approximately 60% confluency, a stage conducive to initiating differentiation, the standard growth medium was replaced with osteogenic induction medium. This specialized medium was formulated to stimulate osteoblast differentiation and subsequent mineralization. It comprised basal medium (DMEM with 10% FBS) supplemented with: ascorbic acid (50 µg/mL), β-glycerophosphate (1 mM), dexamethasone (10 nM), and 1α,25-dihydroxyvitamin D3 (10 nM) (Sigma Aldrich, Buchs, Switzerland). These components collectively promote osteogenesis by providing essential cofactors, phosphate sources, and signaling molecules that activate key transcription factors and signaling pathways involved in bone matrix deposition. The medium was replenished every two days to maintain consistent induction conditions and nutrient availability.

After two weeks of induction, the plates were analyzed for mineralization, a hallmark of osteoblast differentiation. The extent of in vitro calcium deposition was quantified using Von Kossa (VK) staining, which visualizes calcium phosphate deposits, and the presence of mature osteocytes with calcium deposits was assessed using Alizarin Red (AR, Sigma Aldrich, Buchs, Switzerland) staining, which specifically stains calcium ions at pH 4.2.

For VK staining, the medium was carefully aspirated from the wells, and the cells were rinsed three times with PBS to remove residual medium components. Following washing, cells were fixed for 5 min in 10% neutral buffered formalin, preserving cellular structure and preventing further metabolic activity. The formalin was then removed, and the cells were washed three times with deionized water to eliminate residual fixative agent. Subsequently, the cells were stained with a 5% aqueous silver nitrate (AgNO_3_, Sigma Aldrich, Darmstadt, Germany) solution, which reacts with calcium phosphate deposits. The plates were then exposed to UV light for 1 h, facilitating the reduction of silver ions to metallic silver at the sites of calcium deposition, resulting in a dark precipitate. Following UV exposure, the plates were washed three times with water to remove unbound silver. Finally, a 5% aqueous sodium thiosulfate solution (Applichem, Darmstadt, Germany) was added for 2 min to remove unreacted silver nitrate. The cells were then washed three times with water, and the samples were examined under a light microscope for the presence of black clusters, indicative of calcium phosphate deposits.

For AR staining, the working solutions were warmed to 37 °C until they became completely liquid and transparent, ensuring optimal staining conditions. The medium was aspirated, and the wells were washed with deionized water. Cells were then fixed with formalin at ambient temperature, preserving cellular morphology. Following fixation, cells were rinsed with deionized water to remove residual fixative agent. Subsequently, cells were stained with AR solution for 15 min with gentle agitation, protected from light, allowing for specific staining of calcium ions. The stain was then removed, and the cells were rinsed twice with deionized water to remove unbound dye. Microscopic imaging was performed to visualize the presence and distribution of AR staining, indicating areas of calcium deposition and mature osteocyte formation. Pictures were taken using an inverted microscope (Olympus IX81).

### 2.7. Collagen Induction and Quantification in Fibroblast Cultures

To quantitatively assess collagen production by the various fibroblast cell sources, a standardized induction and quantification protocol was employed. Cells were seeded in 12-well culture plates at a viable density of 3 × 10^3^ cells/cm^2^, ensuring a consistent starting cell population for comparative analysis. Cultures were maintained in fibroblast proliferation medium until they reached 100% confluency, a stage at which cells exhibit robust metabolic activity and responsiveness to induction stimuli.

To stimulate collagen synthesis, the culture medium was supplemented with 10^−4^ M vitamin Cp (phosphorylated ascorbic acid, Merck, Darmstadt, Germany), a crucial cofactor for prolyl and lysyl hydroxylases involved in collagen biosynthesis. The induction medium was replenished every two days for one week to maintain consistent vitamin C levels and ensure sustained collagen production.

Following the one-week induction period, the treated cell populations were washed with PBS to remove residual medium components. Cells were then fixed with −20 °C methanol (Fluka, Buchs, Switzerland) for 10 min, preserving the synthesized collagen matrix and cellular structure. After fixation, cells were rinsed three times with PBS to eliminate residual methanol. Samples were either stored at −80 °C for long-term preservation or immediately processed for total collagen analysis.

Sirius Red staining, a well-established method for quantifying collagen content, was employed as described previously [45]. This dye specifically binds to collagen fibers, allowing for colorimetric quantification of collagen deposition. For each sample, 100 µL of dissolved Sirius Red stain (dissolution was made by the addition of 0.1 M NaOH and incubated for 30 min at RT), which had been extracted from the fixed cell layers, were transferred to a new 96-well microplate. Absorbance measurements were performed at a wavelength of 560 nm using a spectrophotometer (Varioskan LUX multimode plate reader, Thermo Fisher Scientific, Waltham, MA, USA), where the absorbance value is directly proportional to the amount of collagen present. This colorimetric quantification allowed for a precise and comparative assessment of collagen production across the different fibroblast cell sources. The data were analyzed using the Skanit-RE software V5.0 (Thermo Fisher Scientific, Waltham, MA, USA).

### 2.8. DED Model for Assessing the Keratinocyte-Structuring Potential of Various Fibroblast Sources

To evaluate the capacity of various fibroblast sources to support keratinocyte organization and stratification, a DED model, adapted from MacNeil et al., was utilized [46]. This model provides a physiologically relevant three-dimensional scaffold for studying epithelial–mesenchymal interactions. DED preparations were generated from abdominoplasty skin tissues, yielding fragments of 1–2 cm^2^.

Briefly, skin fragments were incubated in 1 M NaCl (Bichsel, Unterseen, Switzerland) at 37 °C for up to 72 h. This high-salt treatment selectively disrupts the dermal–epidermal junction, facilitating the delicate separation of the epidermis from the dermis using forceps, while preserving the dermal extracellular matrix integrity. The resulting dermal preparations were then decellularized, removing residual cellular components while maintaining the structural framework of the dermis. These DED preparations were thoroughly washed multiple times with PBS to remove residual NaCl and cellular debris and stored at 4 °C in PBS until use, ensuring hydration and preventing degradation.

To prepare the DEDs for cell seeding and to equilibrate the matrix environment, samples were transferred into 12-well plates containing 1 mL of keratinocyte proliferation medium (DMEM and Ham’s F12 (Merck, Darmstadt, Germany) with a 3:1 proportion, with 20 µg/mL gentamycin (Lausanne University Hospital, Lausanne, Switzerland), 0.14 nM cholera toxin (Lubio Science, Zurich, Switzerland), 400 ng/mL hydrocortisone (Pfizer, New York, NY, USA), 8.3 ng/mL EGF (Merck, Darmstadt, Germany), 832.2 µM L-glutamine, 0.12 U/mL insulin (Novo Nordisk Pharma, Bagsværd, Danemark), and 10% FBS) and incubated at 37 °C under 5% CO_2_ for 2 h. This pre-incubation step ensured optimal hydration and nutrient availability for subsequent cell seeding.

Various cell suspension formulations, designed to achieve different fibroblast-to-keratinocyte or ASC-to-keratinocyte ratios, were prepared. These ratios were selected to mimic physiologically relevant cell densities and to assess the impact of fibroblast or ASC presence on keratinocyte organization. To confine the cell suspensions to the surface of the DED, 8 mm cylindrical glass inserts were carefully placed onto the DED surface. Subsequently, 100 µL of the cell suspension in proliferation medium was dispensed within each insert. This controlled application ensured even cell distribution and direct interaction with the dermal matrix. Each condition was made in replicate, one for Histology and the other to test the viability of the cells.

The DED assay plates were incubated for 3 days, allowing for cellular attachment and initial interactions between the seeded cells and the dermal scaffold. Following this attachment period, the glass inserts were carefully removed, and the DEDs were placed on top of a sterile plastic grid positioned over the culture medium. This configuration established an air-liquid interface, mimicking the physiological environment of the skin and promoting keratinocyte stratification.

After one week of culture, one DED sample was fixed in formalin for 24 h at ambient temperature, preserving cellular and matrix architecture. Fixed samples were then rinsed with PBS and transferred to 70% ethanol at 4 °C until paraffin embedding, facilitating subsequent histological analysis.

To assess cellular viability in situ, the other DED sample was stained with MTT. DED samples were incubated with 0.5 mg/mL MTT in PBS for 2 h at 37 °C. During this incubation, metabolically active cells reduced MTT to insoluble purple formazan crystals. Following incubation, samples were washed twice with PBS to remove unbound MTT, and images were acquired using an iPhone 13 (Apple, Cupertino, CA, USA) to visualize the extent of formazan formation, reflecting cellular viability and distribution.

## 3. Results

### 3.1. Histological Analysis of Human Skin Across Developmental Stages Reveals Distinct Fibroblast Niche Characteristics

The developmental stage of human skin significantly dictates the composition and functional potential of fibroblast populations. In mature adult skin, the epidermis presents a stratified structure, comprising 4–5 layers of keratinocytes culminating in a robust stratum corneum, which serves as a critical barrier against environmental insults. The dermis, a complex connective tissue, harbors a diverse array of specialized structures, including hair follicles, sebaceous glands, and a rich network of vasculature and nerves. The basal layer of the epidermis, characterized by a high proliferative capacity, forms intricate interdigitating projections, termed rete ridges, that extend into the dermis. These structures are pivotal in providing mechanical stability and maintaining homeostatic integrity at the dermal–epidermal junction by increasing the surface area for nutrient and metabolite exchange.

**Figure 1:**
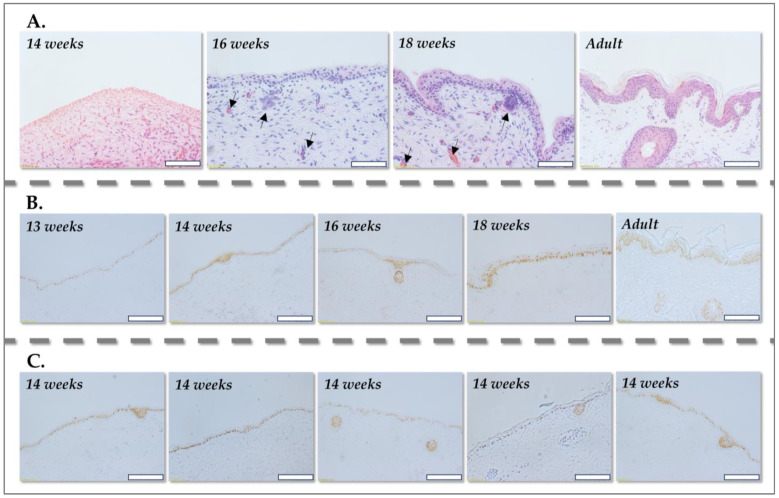
Histology results for skin of various developmental stages. (**A**) H&E staining of human skin at 14–18 weeks of gestation and a control sample from an 80-year-old patient. (**B**) Immunostaining with p63 antibodies (i.e., brown coloration, A4A, Neomarker) showing epidermal/epithelial cell staining for 13–18-week gestation samples and adult abdominal skin. (**C**) Immunostaining with p63 antibodies of 5 different donors of abdominal skin at 14 weeks of gestation. Minor accumulation of basal cells (**A** [14-week] and **C**) and increased dermal accessory cells and vessels were observed (**A** [16, 18 weeks], arrows). Scale bars = 100 µm.

Histological examination using H&E staining revealed a markedly simplified architecture in fetal skin at early gestational stages (10–14 weeks). The dermis is characterized by a relatively loose ECM with sparse cellularity, reflecting the nascent stage of tissue development. Notably, specialized dermal appendages, such as sebaceous glands and hair follicles, are largely absent during this period (Figure 1A).

This simplified dermal structure suggests a more homogenous population of progenitor fibroblasts, potentially offering a distinct advantage for cell-based therapies compared to the heterogeneous fibroblast populations found in mature adult skin. At these early gestational stages, the epidermis is rudimentary, typically comprising a single, undifferentiated cell layer. Immunohistochemical staining with p63, a transcription factor specifically expressed in basal keratinocytes, allowed for precise visualization of this nascent epidermal layer (Figure 1B). Furthermore, the stratification of the epidermis into distinct layers, such as the stratum spinosum and stratum granulosum, is minimal at this stage, resulting in the absence of well-defined rete ridges at the dermal–epidermal junction. This lack of structural complexity reflects the early developmental stage of the epidermis, characterized by limited differentiation and organization.

The initial formation of hair follicle buds, a critical milestone in skin development, is generally observed around 14–16 weeks of gestation, accompanied by the accumulation of mesenchymal cells at the base of these buds, which will eventually form the dermal papilla. Sebaceous gland development commences later, around 18 weeks of gestation. Consequently, in 14-week-old fetal skin, fibroblast niches are relatively sparse, primarily confined to the loosely organized dermal matrix. Furthermore, fibroblast cultures derived from 14-week-old fetal skin consistently exhibit a homogenous population of fibroblastic cells, devoid of detectable p63 expression, confirming the absence of significant keratinocyte contamination. These histological findings are consistent with established literature on skin embryogenesis and development [47].

In stark contrast, adult skin presents a significantly more complex architecture, harboring numerous potential niches for fibroblast populations. These niches can originate from a variety of dermal structures, including sebaceous glands, the perivascular regions of blood vessels, and the mesenchymal components associated with hair follicles (Figure 1A [arrows, 16 weeks, 18 weeks]). Consequently, fibroblast cultures derived from adult skin often exhibit greater heterogeneity, reflecting the diverse origins and functional specializations of these cells within the intricate adult skin microenvironment.

### 3.2. Growth and Stability of Primary Cells and Adipose Stem Cells

#### 3.2.1. Comparative Proliferation Capacity

**Figure 2:**
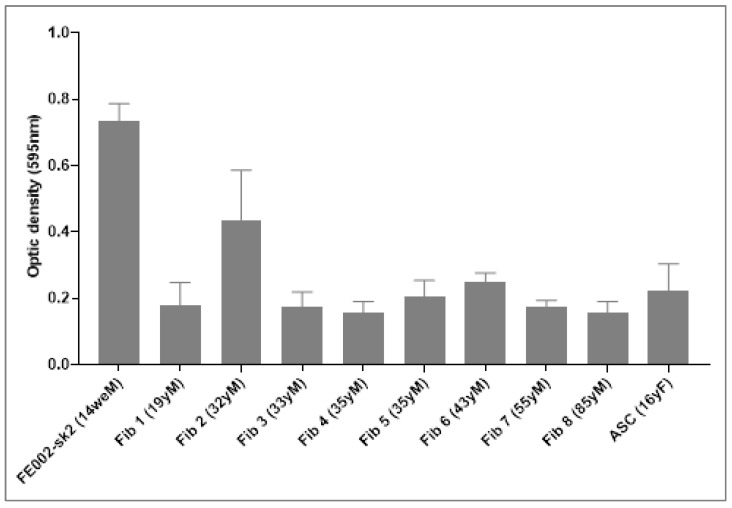
Cell growth at measured by MTT staining for 8 burn patient-derived fibroblast cell sources (Fib 1–Fib 8 cell types), ASCs, and PFF (FE002-SK2 cell type). Proliferation was parallelly assessed at P5–P7 at 10 days of culture. Data are reported for the mean and standard deviation for three independent experiments and in triplicates.

A significant difference in growth potential was observed between PFF and fibroblasts derived from the dermis of eight adult burn patients (Fib 1–Fib 8) ranging in age from 19 to 85 years (Figure 2).

Proliferation assays demonstrated a significantly diminished proliferative capacity in seven out of eight adult fibroblast cultures, exhibiting a four-fold or greater reduction in growth rate compared to PFF (FE002-SK2; Figure 2). This observation underscores the inherent proliferative advantage of PFF compared to adult counterparts cultured in the same way. Similarly, ASCs isolated from a 16-year-old patient displayed reduced proliferation rates, reinforcing the distinct growth kinetics between fetal and adult-derived cells. ASCs were subsequently utilized as a control cell source, particularly for differentiation assays, where their known differentiation potential served as a baseline for comparison.

Intriguingly, no discernible correlation was observed between fibroblast proliferation capacity and donor age within the adult group, as similar growth patterns were consistently observed across a broad age range of 19–85 years (Figure 2). This finding suggests that factors other than chronological age, such as intrinsic cellular properties or environmental influences, may play a more dominant role in determining fibroblast proliferation rates.

Furthermore, when utilizing cells at relatively early passages (P5–P7), minimal differences in proliferation capacity were observed among cultured skin fibroblasts from adult donors [48]. This observation aligns with previous studies indicating that significant disparities in proliferation rates between young and old fibroblasts become more pronounced at later passages [49]. Our findings with fetal fibroblasts are consistent with this observation, highlighting the importance of passage number in influencing fibroblast growth dynamics.

Nonetheless, during the analysis of adult burn patient fibroblast cultures, significant heterogeneity in growth rates was noted. Notably, one patient (Fib 2, 32-year-old donor) exhibited a 50–60% higher proliferation rate compared to the other seven patients (Figure 2). This variability underscores the potential influence of individual patient characteristics, such as wound healing status or systemic factors, on fibroblast proliferation.

#### 3.2.2. Stability and Homogeneity of Primary Fibroblast Sources

**Figure 3:**
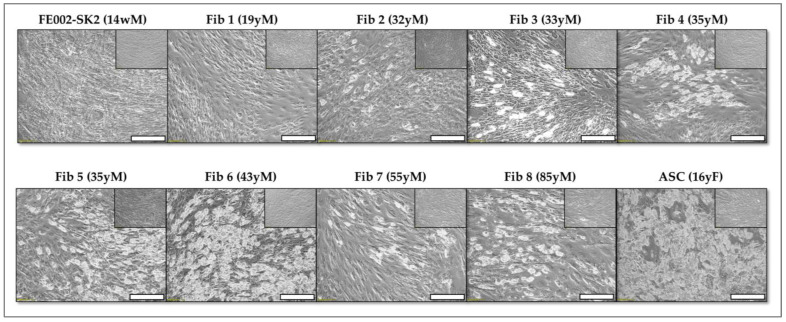
Adipogenicity represented by lipid droplet presence (i.e., round glossy droplets, arrows) in adult fibroblasts (Fib 1–Fib 8) and ASCs for cells in culture at passage level 7 following two weeks of culture in adipogenic differentiation medium. No lipid droplets were observed in FE002-SK2 cells. Scale bars = 200 μm.

Considering the established heterogeneity and differentiation potential of fibroblasts derived from adult sources, we conducted a comparative analysis of these characteristics between adult burn patient fibroblasts and PFF. Specifically, we evaluated the adipogenic differentiation potential in all adult burn patient fibroblast cultures, revealing a broad spectrum of adipogenic capacity, ranging from minimal to substantial lipid accumulation. This observed variability in adipogenic potential did not exhibit any discernible correlation with either patient age or fibroblast proliferation rates (Figure 3).

This lack of correlation suggests that the propensity for adipogenic differentiation in adult burn patient fibroblasts is likely influenced by factors independent of age and proliferative capacity, potentially reflecting intrinsic cellular programming or microenvironmental cues within the burn wound.

The observed heterogeneity in adipogenesis among adult fibroblasts likely stems from the diverse anatomical origins of these cells within the complex architecture of adult skin. In contrast, PFF (FE002-SK2) consistently demonstrated a complete absence of adipogenic differentiation, as no lipid droplets were present. This lack of adipogenesis may be attributed to the limited and less differentiated fibroblast niches present at the 14-week gestational stage, potentially resulting in a more homogenous and stable cell population (Figure 3) [50,51,52,53,54]. ASCs, known for their robust adipogenic potential, exhibited uniform and extensive lipid droplet formation, serving as a positive control for assessing the degree of adipogenic differentiation in adult skin fibroblasts.

We observed that three adult fibroblast samples (Fib 1, 2, and 7) exhibited relatively low adipogenic differentiation, characterized by minimal lipid accumulation. Conversely, the remaining five adult fibroblast samples displayed high differentiation potential, demonstrating substantial lipid droplet formation (Figure 3). This variability underscores the inherent heterogeneity of adult fibroblast populations and its potential implications for cell-based therapies.

The clinical application of adult fibroblasts in burn patient care has been constrained by the prolonged culture time required to achieve sufficient cell numbers for therapeutic interventions. Further investigation is warranted to elucidate whether the diminished proliferation and variable adipogenicity observed in some adult fibroblast samples may impact treatment efficacy when utilizing these cells at different passage numbers. Specifically, it is crucial to determine if these characteristics affect the functional properties of the cells in wound healing. Importantly, all adult fibroblast samples retained their adipogenic differentiation potential over multiple passages in culture (up to passage level 10), indicating a stable phenotype despite extended culture periods.

#### 3.2.3. PFF at 14 Weeks of Gestation Lack Adipogenic and Osteogenic Differentiation Potential

To further substantiate the observed lack of differentiation potential in the clinical PFF cell source (FE002-SK2), an additional four PFF cell cultures were established for research purposes. All primary cell cultures were derived from fetal skin samples at 14 weeks of gestational age and were cultured under identical conditions to ensure experimental consistency.

**Figure 4:**
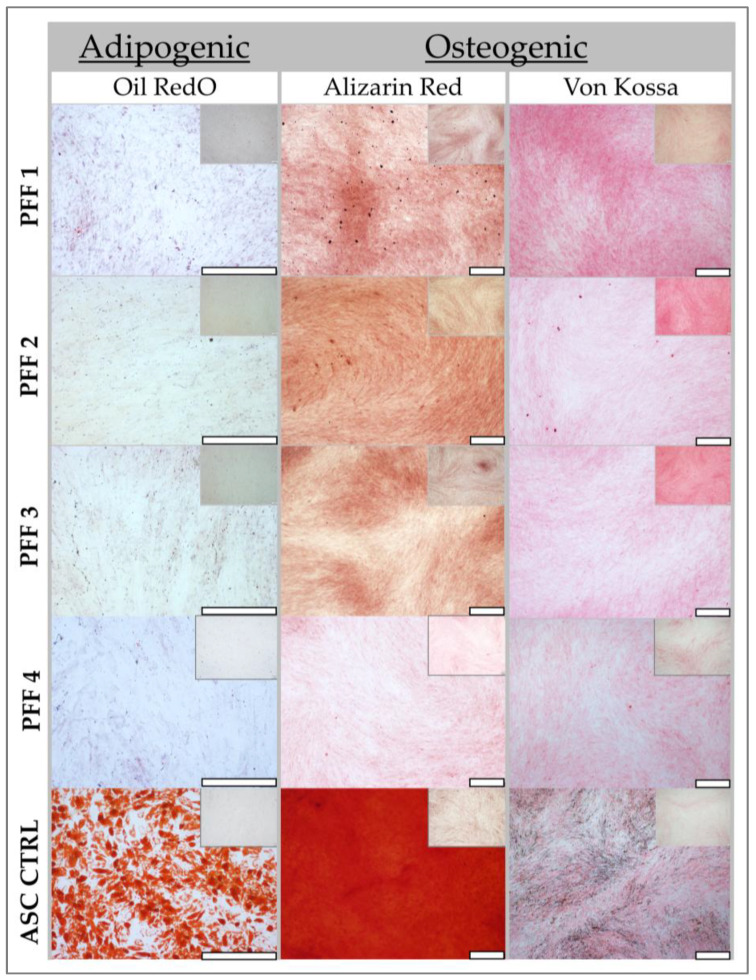
Differentiation potential for adipogenesis and osteogenesis at P7 for PFF 1–4, represented by Oil Red O staining for adipogenesis. ASC controls showed bright red positive staining. Osteogenesis was negative, as shown by lack of intense red coloration for AR or brown deposits for VK staining compared to the ASC positive control with intense staining throughout the cell culture. Photos were paired with non-stained controls (insert, top right corner). Scale bars = 400 μm.

In addition to adipogenesis, the osteogenic differentiation potential of all fibroblast cultures was assessed in a two-dimensional (2D) culture system using two distinct staining methods, allowing for a comprehensive evaluation of their differentiation capacity. Consistent with the initial clinical PFF cell source, none of the four additional PFF cultures exhibited lipid droplet formation or positive staining with Oil Red O, definitively confirming the absence of adipocyte differentiation (Figure 4).

This consistent lack of adipogenesis across multiple PFF cultures reinforces the data on the inherent stability and limited differentiation potential of these cells. The ASC positive control exhibited intense Oil Red O staining, indicative of robust lipid accumulation, and readily observable lipid droplet formation (Figure 4, insert, upper right corners). This observation confirmed the efficacy of the adipogenic induction protocol and served as a benchmark for comparing the differentiation potential of other cell types. Similarly, ASCs displayed strong osteogenic differentiation, characterized by intense AR staining, signifying calcium deposition, and the presence of brown deposits throughout the culture, a hallmark of VK staining following osteogenic induction. These results validated the osteogenic induction protocol and confirmed the differentiation capability of ASCs.

Conversely, none of the PFF cultures exhibited positive staining for osteogenic differentiation. Specifically, no AR coloration, indicative of calcium binding, or brown deposits, characteristic of VK staining, were observed in any of the PFF cultures. These findings suggest that PFFs lack the capacity for osteogenic differentiation under the tested conditions. The cells were serially passaged up to passages 10–12 and subsequently re-evaluated for differentiation potential to assess the stability of this phenotype. Notably, no differentiation was observed in any of the conditions tested for the PFF cultures, confirming the consistent absence of adipogenic and osteogenic differentiation potential over multiple passages.

It is well-established that cellular heterogeneity within adult skin can significantly influence fibroblast differentiation potential. To further investigate this phenomenon, adult skin samples were meticulously dissected to separate the papillary and reticular dermis, representing distinct dermal layers with varying cellular compositions and functions. Fibroblast cultures were then established from each layer using mechanical dissection and outgrowth, avoiding enzymatic digestion to minimize potential alterations in cellular phenotype. Prior research, employing enzymatic digestion and isolating fibroblasts from different anatomical sites, has revealed variations in differentiation capacity, with reticular fibroblasts typically exhibiting higher differentiation potential, likely due to their distinct developmental origins and functional roles.

**Figure 5:**
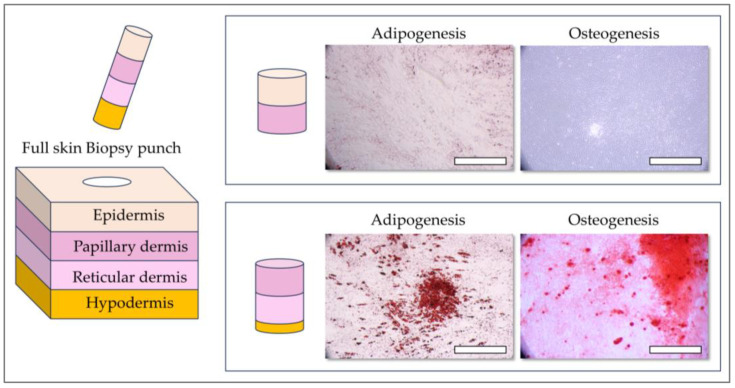
Differentiation potential for adipogenesis and osteogenesis at P3 for adult fibroblasts isolated either from the papillary dermis (i.e., dark pink layer) or from the reticular dermis (i.e., light pink layer). Adipogenesis is visualized by Oil Red O staining. Osteogenesis was shown with intense red coloration for AR. Scale bars = 500 μm.

Consistent with these prior findings, our results demonstrated that papillary fibroblasts exhibited lower differentiation potential for both adipogenic and osteogenic lineages compared to reticular fibroblast populations (Figure 5).

This observation underscores the importance of considering dermal layer specificity when evaluating fibroblast differentiation potential and highlights the impact of cellular heterogeneity on differentiation outcomes.

These findings suggest that, in addition to inter-patient variability, intra-patient heterogeneity within a single skin biopsy can significantly contribute to variations in fibroblast differentiation potential. Specifically, this variability may be influenced by the depth of the dermal layer and its proximity to the hypodermis, which contains distinct cell populations and ECM components that can influence cellular programming.

Papillary dermal fibroblasts, located more superficially within the dermis, consistently exhibited lower overall differentiation potential for both adipogenesis and osteogenesis (Figure 5). This reduced differentiation capacity may be attributed to their distinct microenvironment, potentially characterized by lower fibroblast density and fewer interactions with cell types known to influence differentiation, such as those found in proximity to ASCs in deeper dermal layers.

**Figure 6:**
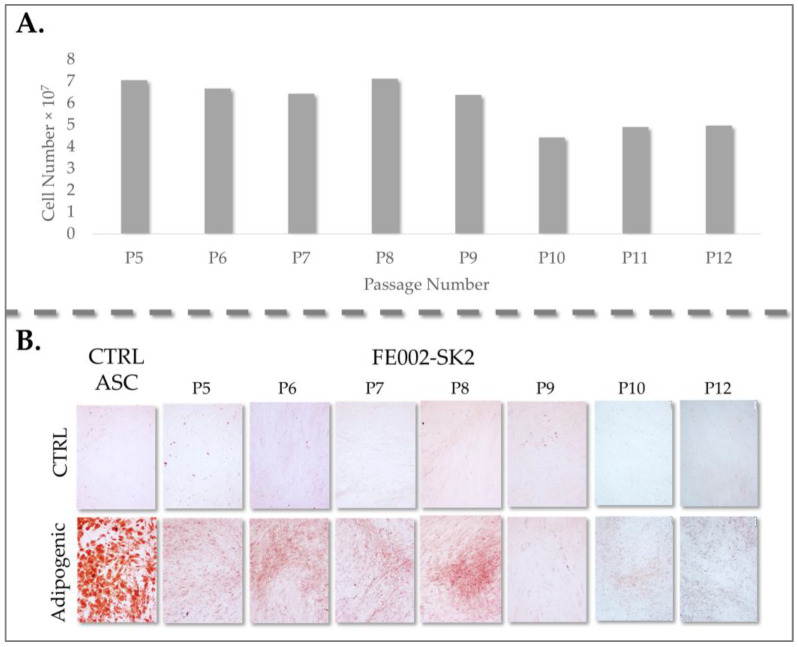
(**A**) FE002-SK2 cell number in function at different passages (P5–P12). (**B**) Cells were placed in adipogenic differentiation for 2 weeks and stained with Oil Red O at different passages, with ASC (P7) as control cells (i.e., first column on left). ASC, adipose stem cells (Scale bars = 400 μm).

Of note, clinical application of these cells has been standardized at passage level 7, a point within the passage range demonstrating optimal and consistent growth characteristics under the defined culture conditions. This selection was based on the observed stability and robust proliferation of the PFF cell source (FE002-SK2) within this passage range. Throughout these passages, the FE002-SK2 cell source consistently maintained its undifferentiated phenotype, evidenced by the complete absence of adipogenic differentiation. This lack of adipogenesis was confirmed by the failure to exhibit Oil Red O staining, contrasting sharply with the intense red staining indicative of lipid accumulation observed in control ASC cultures (Figure 6).

This observation reinforces the stability and limited differentiation potential of the FE002-SK2 cell source, highlighting its suitability for clinical applications where a consistent and undifferentiated fibroblast population is desired.

### 3.3. Comparative Collagen Production of Primary Fibroblasts and Adipose Stem Cells

Our investigations revealed that fibroblast populations derived from 14-week gestational age skin exhibit unique characteristics that differentiate them from fibroblasts obtained from other developmental stages or those isolated from burn patients. A critical distinction lies in the composition of their ECM. Collagen, a major structural component of the ECM, plays a pivotal role in tissue integrity and wound healing. Therefore, we conducted a comparative analysis of collagen content and expression in cultured PFF (FE002-SK2) against adult fibroblasts and ASCs. To ensure a valid comparison, cells were cultured to similar confluency at the same passage number, minimizing variability due to cell density and passage-related effects.

**Figure 7:**
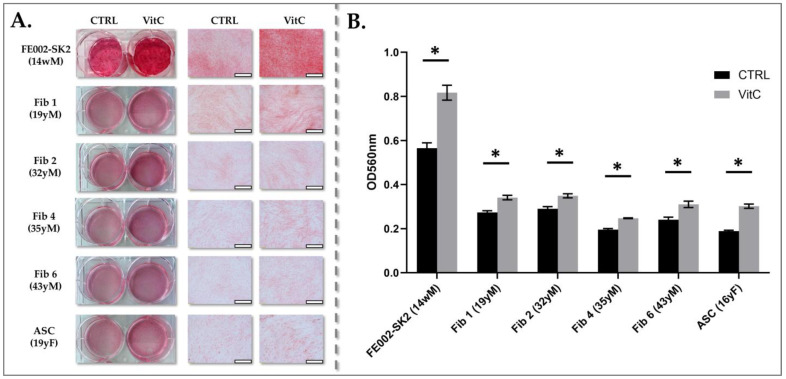
(**A**) Collagen production evidenced by Sirius Red staining of FE002-SK2 fibroblasts, adult fibroblasts (Fib 1, 2, 4, and 6) and ASCs. Cells at the same confluency level were stained before (control) and after vitamin C stimulation for collagen deposition. Scale bars = 500 μm. (**B**) Collagen production assessed by OD_560_ measurements of extracted Sirius Red. Significant differences with control values were evidenced with an asterisk *.

Collagen deposition was quantified using Sirius Red staining, a technique that specifically stains collagen fibers, both under basal conditions and following one week of vitamin C stimulation upon reaching confluency (Figure 7A).

Vitamin C, a crucial cofactor in collagen biosynthesis, was used to assess the cells’ capacity for enhanced collagen production under stimulatory conditions. To ensure accurate comparison of collagen deposition, cell counts were performed in triplicate for each cell type. PFF (FE002-SK2) exhibited a mean cell count of 3.70 ± 0.10 × 10^5^ cells/cm^2^, indicating a consistent cell density. Adult fibroblast cell types displayed an average cell count of 3.48 ± 0.85 × 10^5^ cells/cm^2^, reflecting a slightly wider range of cell densities. ASCs showed a mean cell count of 3.63 ± 0.25 × 10^5^ cells/cm^2^, demonstrating a relatively uniform cell distribution. Sirius Red staining, which specifically binds to collagen, revealed low to moderate levels of collagen in all adult fibroblast and ASC primary cultures, both under basal conditions and following vitamin C stimulation. In contrast, FE002-SK2 fibroblasts exhibited moderate to high collagen staining at comparable confluency, as evidenced by both visual inspection of the stained cultures and quantitative cell counts (Figure 7A).

Semi-quantitative analysis of collagen content, derived from the intensity of Sirius Red staining, revealed that vitamin C stimulation significantly enhanced collagen deposition in all fibroblast and adipocyte cultures tested (Figure 7B). Notably, FE002-SK2 fibroblasts demonstrated a 2–3-fold higher collagen content compared to other cell types. This substantial difference was readily apparent upon macroscopic observation of the Sirius Red-stained cultures (Figure 7A), highlighting the superior collagen-producing capacity of PFFs. Furthermore, collagen content in adult fibroblasts did not exhibit any significant age-related dependency, as overall collagen levels remained relatively consistent between baseline and vitamin C-stimulated cultures (Figure 7B). This observation suggests that the capacity for collagen production in adult fibroblasts is not significantly influenced by donor age, at least within the tested conditions.

### 3.4. Keratinocyte-Structuring Functions of Primary Fibroblasts and ASCs

Fibroblasts are integral to the orchestration of human skin formation, exerting critical regulatory influence on processes such as basement membrane construction and ECM organization [55,56,57,58,59]. To investigate the impact of PFF (FE002-SK2) on epidermal development, we utilized the DED model, a physiologically relevant three-dimensional scaffold, to co-culture these cells with keratinocytes. Previous studies employing neonatal fibroblasts and keratinocytes for wound healing applications have reported optimal results with a fibroblast-to-keratinocyte ratio of 9:1, reflecting the importance of adequate fibroblast support for epidermal stratification and differentiation [35,36].

**Figure 8:**
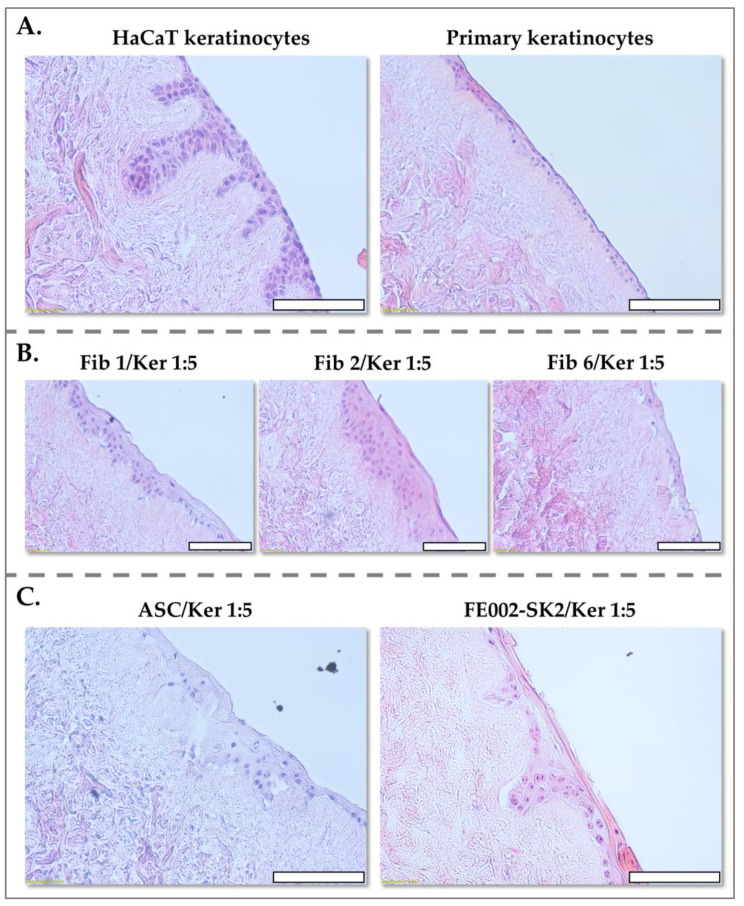
Formation of skin equivalents on DED with various ratios of cell suspensions from different cell types. (**A**) HaCaT cells and primary keratinocytes seeded alone. (**B**) Fibroblasts from three burn patients (Fib 1, 2, and 6 [19, 32 and 43 years, respectively]) and primary keratinocytes seeded at a 1:5 ratio. (**C**) ASCs or FE002-SK2 and primary keratinocytes seeded at a 1:5 ratio. Scale bars = 100 μm.

Our findings demonstrated that FE002-SK2 fibroblasts exhibit a remarkably potent effect in co-culture with keratinocytes within the DED model. Notably, a significantly lower fibroblast-to-keratinocyte ratio of 1:5 was sufficient to facilitate efficient epidermal development (Figure 8, FE002-SK2/Ker 1:5).

This observation suggests that FE002-SK2 fibroblasts possess enhanced signaling capabilities or ECM production, enabling robust keratinocyte organization and stratification even at lower cell densities.

Primary keratinocytes cultured alone on the DED substrate formed a thin, poorly organized layer, lacking a distinct epidermal/dermal interface (Figure 8A). This observation underscores the critical role of fibroblasts in providing structural and signaling cues necessary for proper epidermal stratification and organization.

Co-cultures utilizing adult burn patient fibroblasts at a 1:5 ratio resulted in the formation of multi-layered keratinocyte structures. However, these structures exhibited varying thicknesses and disorganized keratinization, indicating suboptimal epidermal differentiation (Figure 8B). In contrast, co-cultures of PFF (FE002-SK2) with adult primary keratinocytes, cultured in serum-free media at a 1:5 ratio, demonstrated the formation of well-defined epidermal structures (Figure 8C). These co-cultures exhibited a single to double layer of keratinocytes, a distinct stratum corneum, and evident rete ridge formation as early as one week post-culture (Figure 8C). This rapid and organized epidermal development highlights the superior capacity of FE002-SK2 fibroblasts to promote keratinocyte stratification and differentiation.

ASCs, when co-cultured with keratinocytes at a 1:5 ratio, yielded results comparable to those observed with adult fibroblasts, characterized by multi-layered keratinocyte structures but lacking significant keratinocyte penetration into the dermal structure (Figure 8C). This observation suggests that ASCs, while capable of supporting keratinocyte proliferation, do not effectively facilitate the formation of a well-organized epidermal/dermal interface.

Control cultures using HaCaT transformed keratinocytes demonstrated significant keratinocyte invagination into the dermal structure, a characteristic reminiscent of normal skin biopsies (Figure 8A). However, it is noteworthy that previous studies employing bi-layered living cellular models with normal fibroblasts and keratinocytes have rarely demonstrated such clear keratinocyte orientation and rete ridge formation within the dermal substrate, a hallmark of normal skin architecture. The robust rete ridge formation observed in FE002-SK2 co-cultures highlights the unique ability of these fetal fibroblasts to recapitulate key aspects of normal skin architecture in vitro.

## 4. Discussion

### 4.1. Key Complementary Contributions of Fibroblasts and Keratinocytes in Burn Wound Healing

Burn injuries represent a formidable global health challenge, affecting over 11 million individuals annually. These injuries inflict profound physical and psychological trauma, contributing to high morbidity and mortality rates, as well as substantial long-term economic burdens [17]. Historically, a disproportionate percentage (up to 90%) of burn injuries have occurred in low- and middle-income countries, with children and the elderly being particularly vulnerable. However, the escalating frequency of global conflicts may alter this epidemiological trend, further necessitating the development of robust and readily deployable cell-based therapies for burn wound and scar management, especially in resource-constrained environments [12,15]. Cell therapies hold immense promise, surpassing conventional treatment modalities by stimulating intrinsic tissue regeneration mechanisms. These therapies have demonstrated remarkable efficacy in promoting angiogenesis, epithelialization, granulation tissue formation, and collagen deposition, while also exerting potent anti-oxidative and anti-inflammatory effects that mitigate apoptosis [1,2,3,4,5,6,7].

Burn injuries are dynamic and progressive, often characterized by sustained inflammation, compromised microvascular perfusion, and eventual tissue necrosis [2,3]. The stasis phase, an intermediate stage of burn injury, represents a critical therapeutic window. Timely and effective interventions during this phase are paramount to prevent burn progression and optimize wound healing outcomes. The overarching goal is to develop innovative therapeutic strategies that effectively attenuate inflammation, stimulate regenerative processes, and ultimately minimize scar formation [45].

It is well-established that the combined application of keratinocytes and fibroblasts yields superior clinical outcomes in burn patients [2,60]. This enhanced efficacy is attributed to the synergistic interplay between these cell types, supported by both developmental and clinical research.

The synergistic interaction between fibroblasts and keratinocytes is believed to be mediated by the crucial roles of ECM synthesis and soluble factor signaling in supporting keratinocyte graft development [32,33,34]. These cell types engage in intricate paracrine signaling pathways, facilitating the restoration of normal tissue architecture. Therefore, early incorporation of fibroblasts in burn therapy can significantly contribute to the formation of a suitable granulation tissue and wound bed, creating an optimal microenvironment for keratinocyte attachment and differentiation [32,33,34].

The inherent heterogeneity of fibroblast populations within burn wounds underscores the importance of careful cell source selection for optimal therapeutic outcomes. Varkey et al. demonstrated in 2014 the significant influence of fibroblast origin by comparing superficial dermal fibroblasts to those derived from deeper dermal layers [61]. Their study, utilizing a tissue-engineered skin model, revealed that superficial dermal fibroblasts, when combined with keratinocytes, exhibited enhanced dermo-epidermal adhesion and anchoring protein expression, resulting in the formation of a continuous epidermis with improved barrier function. Furthermore, superficial fibroblast-keratinocyte combinations demonstrated higher expression levels of key epidermal proteins, such as keratin-5 and E-cadherin, and exhibited more robust basement membranes with increased levels of laminin-5, nidogen, and collagen type VII compared to combinations using deep dermal fibroblasts [61].

Our findings further corroborate the influence of fibroblast origin by demonstrating that papillary dermal fibroblasts (superficial dermal fibroblasts) exhibit a reduced differentiation potential toward adipogenesis and osteogenesis compared to deeper dermal fibroblasts. These distinctions highlight the necessity for tailored fibroblast selection in therapeutic applications.

Current tissue-engineering strategies often employ heterogeneous populations of dermal fibroblasts, which can exhibit significant variability in differentiation capacity across different patients. Despite their importance, the use of patient-derived fibroblasts presents several challenges [30]. The prolonged culture and expansion time required for fibroblast preparation can significantly delay treatment initiation. Furthermore, adult fibroblasts are known to exhibit fibrotic tendencies during wound healing, and this propensity can vary depending on patient age and individual healing characteristics [31].

### 4.2. Manufacturing Considerations for Autologous Cutaneous Cell Therapies

In burn care, autologous skin grafts remain the gold standard, offering optimal biocompatibility and minimizing immunogenic responses. However, their application is often limited in cases of extensive burns, where sufficient donor skin is unavailable, or when the wound bed is heavily contaminated, necessitating temporary wound coverage. This is commonly achieved using human cadaver skin, porcine xenografts, tilapia skin, their acellular derivatives, or other dermal substitutes, each with varying degrees of efficacy and limitations [2,21].

CEAs and CDEAs have been integrated into routine patient care in specialized centers with accredited cell culture facilities and trained personnel [1,2]. However, CEA production typically requires 2–3 weeks, while the inclusion of fibroblasts in CDEA extends the manufacturing process to approximately 6 weeks. Although these autologous approaches offer advantages over split-thickness skin grafts, they are characterized by prolonged production times, increased fragility, and elevated costs, particularly outside specialized hospital settings equipped with good manufacturing practices (GMP)-compliant manufacturing capabilities [2].

From a product composition standpoint, CEA consists solely of cultured keratinocytes derived from the patient, while CDEA incorporates both patient-derived keratinocytes and fibroblasts. A major challenge associated with these approaches is the protracted time required to generate sufficient cells for timely patient treatment, potentially delaying critical interventions [4,5]. Current clinical practice typically reserves CDEA use for patients with burns exceeding 70% TBSA, allowing sufficient time for the production of autologous skin fibroblasts. A significant advantage of combining keratinocytes and fibroblasts in CDEAs is the improved final skin reconstruction outcome, characterized by reduced tissue contraction and enhanced aesthetic appearance. This is particularly beneficial for challenging anatomical sites, such as joints and the neck/face region, where minimizing skin contracture is paramount [2,45]. However, the source and manufacturing methods of fibroblasts are crucial considerations, as fibroblast characteristics can vary significantly depending on the harvest site, tissue origin, donor age, and manufacturing protocols [30,31].

Recognizing the significant time constraints associated with CEA and CDEA manufacturing, recent advancements have enabled more rapid expansion of patient keratinocytes without the use of feeder layers, reducing the time to cell availability to approximately 10–12 days [62,63]. This expedited production timeline facilitates earlier initiation of patient therapy, potentially improving clinical outcomes. Furthermore, exploring the potential cellular differences between adult and fetal fibroblasts may offer valuable insights into the mechanisms underlying scar-free tissue regeneration, a phenomenon well-documented in vitro and in vivo in both human and animal models [64,65,66,67,68,69,70,71].

Our results demonstrated that PFFs exhibit a significantly higher proliferative capacity compared to adult fibroblasts, coupled with an enhanced ability to synthesize collagen. These findings align with previous studies describing the intrinsic advantages of fetal fibroblasts, including their greater proliferative potential, accelerated migration, and enhanced tissue regeneration capabilities. Additionally, fetal fibroblasts have been reported to exhibit a distinct ECM remodeling profile, characterized by increased synthesis of type III collagen, a key component of scarless healing, and a more balanced expression of inflammatory cytokines, favoring scarless wound healing. The PFF cells have been locally used in the clinic for more than 20 years and no adverse events have been reported. These types of cell sources do not have transplantation antigens and have not shown any deleterious immune responses when transplanted to patients [21]. These properties underscore the potential of PFF in regenerative medicine and clinical applications [38,39,40,41,42,43,44,45].

Independently from the tissues used for cell source establishment, there are also implications of cell doses and ratios of cell types. Notably, previous studies utilizing autologous fibroblasts identified a 9:1 fibroblast-to-keratinocyte ratio as optimal for epidermal formation [35,36]. However, our results demonstrate that PFF (FE002-SK2 cells) effectively support epidermal development at a significantly lower ratio of 1:5. Co-cultures of FE002-SK2 fibroblasts with keratinocytes led to the formation of well-structured epidermal layers, characterized by distinct rete ridges and a stratified keratinocyte layer, within one week. Compared to adult fibroblasts or ASCs, FE002-SK2 fibroblasts exhibited superior epidermal organization.

The utilization of PFF for the development of allogeneic formulations could eliminate the need for surgical procedures to obtain autologous skin and circumvent prolonged cell culture. Furthermore, these fetal cells promote superior epidermal structure compared to autologous cells, even at a lower fibroblast-to-keratinocyte ratio of 1:5. A spray or gel formulation incorporating this optimized cell ratio could offer a promising solution for enhancing tissue regeneration and wound healing. This approach may facilitate easier application while ensuring optimal skin repair, potentially optimizing clinical practice.

### 4.3. The Promising Approach of Skin Progenitor Cells for Mediating Scarless Wound Healing

For high-capacity cell expansion and the establishment of robust cell banks for burn and wound management, FE002-SK2 have been utilized in clinical settings for several decades. These cells have been primarily employed in clinical trials for severe burn patients, demonstrating their therapeutic potential, although they have not yet been developed as a commercially available living cell product. A significant advantage of this cell source is its immediate availability for patient use. Derived from a single organ donation, large master and working cell banks can be established under current Good Manufacturing Practice (cGMP) conditions, facilitating rapid clinical trial initiation and streamlining the translational pipeline [38]. It has been observed that the regenerative properties of fetal fibroblasts can vary depending on their developmental age, influenced by factors such as differences in ECM composition and cell migratory capabilities [43].

As demonstrated by Ersch’s 1999 publication [47] and our histological analyses of skin at different developmental stages, the unique characteristics of PFFs, particularly those derived from tissue before 14 weeks of gestation, are closely associated with the simplified, less structured microenvironment of developing tissue. Unlike adult skin, where fibroblasts originate from a diverse array of niches, fetal skin at these early stages lacks these complex structures. This absence of specialized microenvironments and accessory cells results in a more homogeneous fibroblast population, which may contribute to their increased proliferative capacity, enhanced ECM remodeling potential, and reduced senescence compared to adult fibroblasts.

Other stem cell sources, such as those derived from neonatal foreskin, umbilical cord, Wharton’s Jelly, placenta, and adipose tissue, typically necessitate multiple organ donations to establish cell banks of comparable size, introducing potential variability and logistical complexities [17,18,19,22]. Therefore, identifying cell types that can be consistently produced under standardized procedures, with high cell yields to ensure long-term availability for large-scale processing, is crucial for advancing their therapeutic potential. A key advantage of dermal fibroblast progenitors is their inherent homogeneity and stability. These cells, derived from a specific developmental stage, exhibit a uniform population, minimal differentiation potential, and high resistance to oxidative stress and cryopreservation [39,40,41,42].

Progenitor cells possess the intrinsic capacity to guide scarless wound healing across diverse wound types, including burns, pressure ulcers, and chronic ulcers. These cells provide an inherent collagen network that serves as a scaffold for the regeneration of cutaneous tissues, promoting organized tissue repair. To ensure the consistent quality and stability of cell sources used in clinical settings, it is crucial to compare them with other skin biopsies obtained from the same gestational age and processed using identical manufacturing protocols. This approach enables the creation of cell banks for rigorous analysis and stringent quality control. The stability and overall quality of cell cultures are assessed based on factors such as growth capacity, lack of differentiation potential, uniformity, and consistency. Histological examination of the tissues used to initiate cell cultures has revealed that the uniform quality of the resulting cultures may be linked to the absence of specific cellular niches that are present in tissues from younger or older individuals.

Several cell populations, including those derived from fetal and neonatal sources, have been utilized in clinical settings for burn and wound treatment [10,11,12,13,14,15,16,17,18,19,20,21]. These cell populations often exhibit characteristics of stem cells, such as the capacity for differentiation. While this inherent multipotency can be advantageous for certain applications, it also presents challenges in the clinical setting. The potential for stem cells to differentiate into various cell types, while beneficial for tissue regeneration, can also pose a risk of uncontrolled differentiation. Maintaining the desired cell phenotype in culture requires careful control of the growth environment, including the use of specific growth factors and signaling molecules [21,29,37,62,72,73]. However, once these cells are implanted into the patient, they may be influenced by the diverse array of growth factors and cytokines present in the wound microenvironment, potentially leading to unexpected differentiation and compromising therapeutic outcomes.

Our results have emphasized the importance of source tissue and manufacturing procedures in ensuring consistent cell quality. Therefore, the use of stable progenitor cell sources that exhibit limited differentiation potential offers significant advantages. The validation from separate organ donations has shown that cells manufactured under standardized procedures consistently produce cells that do not differentiate. This difference seen by others where cell sources differentiated easily may be due to gestational period differences in source tissue or also to manufacture differences and raw materials used [47,53,62,72,73].

These cells provide a reliable and consistent foundation for early treatment protocols in patients requiring tegument coverage, offering a promising avenue for scarless wound healing and improved clinical outcomes.

### 4.4. Study Limitations and Research Perspectives

While this study provides compelling evidence for the potential of PFFs in promoting skin regeneration, it is important to acknowledge certain limitations that warrant consideration.

Firstly, the in vitro nature of several key experiments, particularly the DED model, limits the direct translation of these findings to the complex in vivo environment of a burn wound. While the DED model offers a valuable tool for studying cell–cell interactions, it does not fully recapitulate the intricate interplay of factors such as vascularization, immune responses, and systemic influences that are present in clinical burn injuries.

Secondly, although we demonstrated the superior performance of PFFs compared to adult fibroblasts and ASCs, the study focused primarily on a limited number of cell types. Further research is needed to compare PFFs with other potential cell sources, such as those derived from neonatal foreskin or umbilical cord, to comprehensively evaluate their relative efficacy.

Thirdly, while we observed a consistent lack of differentiation potential in PFFs, further investigation is warranted to fully characterize their response to a broader range of differentiation stimuli and to explore the molecular mechanisms underlying their phenotypic stability. Understanding the precise factors that maintain their progenitor state is crucial for ensuring their safe and predictable application in regenerative medicine.

Fourthly, the study’s focus on collagen production and epidermal development provides valuable insights into the regenerative potential of PFFs. However, further research is necessary to comprehensively assess their impact on other critical aspects of wound healing, such as angiogenesis, inflammation modulation, and ECM remodeling.

Building upon these findings, future research should focus on further elucidating the molecular mechanisms underlying the superior performance of PFFs in supporting skin regeneration. This could involve investigating the specific signaling pathways activated by PFFs and their interactions with keratinocytes, including detailed analyses of growth factor secretion, cell surface receptor expression, and ECM protein deposition. Additionally, exploring the potential of PFFs in combination with other biomaterials, such as hydrogels or scaffolds, could enhance their therapeutic efficacy and expand their clinical applications, providing a more versatile platform for cell delivery and tissue regeneration.

Furthermore, pre-clinical studies in animal models of burn injury are warranted to validate the findings observed in vitro and comprehensively assess the in vivo regenerative potential of PFF-based therapies. These studies should evaluate key parameters such as wound healing rates, scar formation, and overall functional recovery, including assessments of skin elasticity, tensile strength, and sensory function.

Finally, rigorous safety and efficacy assessments will be crucial before translating these findings into clinical practice. This will involve conducting well-designed clinical trials to evaluate the safety and efficacy of PFF-based therapies in human patients with burn injuries. These clinical trials should incorporate appropriate controls, standardized outcome measures, and long-term follow-up to ensure patient safety and demonstrate the clinical benefits of PFF-based therapies. By addressing these critical research areas, innovative cell-based therapies that harness the regenerative potential of PFFs may improve outcomes for patients with burn injuries in the future.

## 5. Conclusions

This study has demonstrated the unique characteristics and significant regenerative potential of PFF derived from 14-week gestational age fetal skin. These PFF populations were shown to be homogeneous and consistent under defined manufacturing settings, exhibiting a stable phenotype and a notable lack of differentiation potential, specifically avoiding differentiating into adipocytes or osteocytes. This inherent stability offers a distinct advantage for cell-based therapies, mitigating the risk of unwanted cell fate changes.

Furthermore, compared to adult fibroblasts, PFFs exhibited distinct and advantageous functional properties. Notably, PFFs displayed robust collagen production, a crucial factor for ECM remodeling and tissue repair, and demonstrated a remarkable capacity to support epidermal development in a DED model, even at a low fibroblast-to-keratinocyte ratio of 1:5. This efficient support of epidermal organization, with the formation of well-structured epidermal layers including rete ridges, highlights the superior regenerative capacity of PFFs.

The observed differences in differentiation potential, collagen production, and co-culture behavior underscored the unique properties of PFFs compared to adult primary skin fibroblasts and ASCs. These findings strongly suggest that PFFs may offer significant advantages in tissue engineering applications, particularly in the development of advanced skin substitutes and innovative wound healing strategies. Their inherent stability, high collagen production, and potent support of epidermal development make them a promising cell source for regenerative medicine.

Further investigation into the underlying mechanisms driving these unique characteristics, including a comprehensive analysis of specific signaling pathways, ECM components, and cellular interactions, is warranted to fully elucidate the therapeutic potential of PFFs in cutaneous regenerative medicine and to optimize their application in clinical settings.

## Data Availability

The data presented in this study are available within the article files.

## Abbreviations

AD-MSC	adipose-derived mesenchymal stem cells
AR	Alizarin red
ASC	adipose stem cells
BM-MSC	bone marrow-derived mesenchymal stem cells
CDEA	cultured dermal–epidermal autograft
CEA	culture epithelial autograft
CHUV	Centre Hospitalier Universitaire Vaudois
cGMP	current good manufacturing practices
DED	de-epidermized dermis
DMEM	Dulbecco’s modified Eagle medium
ECM	extracellular matrix
FBS	fetal bovine serum
FE002-SK2	clinical grade dermal progenitor fibroblast source
GMP	good manufacturing practices
H&E	hematoxylin and erythrosin
hPL	human platelet lysate
ITS	insulin-transferrin-selenium
MTT	tetrazolium salt, bromide de 3-(4,5-dimethylthiazol-2-yl)-2,5-diphenyl tetrazolium
NGS	normal goat serum
PBS	phosphate-buffered saline
PFF	progenitor fetal fibroblasts
TBSA	total body surface area
USA	United States of America
UV	ultraviolet
VK	von Kossa
